# Cross-sectional evaluation of exposure to ozone, nitrogen dioxide, and particulate mass levels on circulating immune markers in women in the California Teachers Study

**DOI:** 10.21203/rs.3.rs-7566245/v1

**Published:** 2025-09-30

**Authors:** Emily L. Cauble, Michael J. Kleeman, Yusheng Zhao, Meredith Franklin, Mandy Yao, Emma S. Spielfogel, Tarik Benmarhnia, James V. Lacey, Mitchell S. V. Elkind, Juan Zhao, Larry Magpantay, Otoniel Martinez-Maza, Sophia S. Wang, Marta Epeldegui

**Affiliations:** Beckman Research Institute; University of California, Davis; University of California, Davis; University of Toronto; University of Toronto; Beckman Research Institute; Scripps Institution of Oceanography; Beckman Research Institute; American Heart Association; American Heart Association; University of California Los Angeles; University of California Los Angeles; Beckman Research Institute; University of California Los Angeles

**Keywords:** Air pollution, Immune markers, Human population, Macrophage activation, Pro-inflammatory response

## Abstract

Exposure to ambient air pollutants, specifically ozone (O_3_), nitrogen dioxide (NO_2_), ultrafine, fine or coarse particulate matter (PM_0.1_, PM_2.5_, and PM_10_), has been linked to a number of adverse health outcomes, including cardiovascular disease. Changes in immune response may be a key mechanism underlying these effects. Within the California Teachers Study cohort, we conducted a cross-sectional analysis of 1,898 women to assess the associations between exposure to O_3_, NO_2_, PM_0.1_, PM_2.5_, and PM_10_ and 15 immune markers measured from serum samples collected in 2015. Daily residential exposures to O_3_, NO_2_, PM_0.1_, PM_2.5_, and PM_10_ were estimated by a validated chemical transport model and averaged over 12-, 3-, and 1-month periods prior to blood draw. Fifteen immune markers (categorized as quartiles) were estimated per interquartile range (IQR) of air pollutant exposures using multivariable ordinal logistic regressions adjusted for age, body mass index, and respective pollutants. Immune markers were also grouped into immune pathways (pro-inflammatory/macrophage activation, B-cell activation, and T-cell activation). After applying Bonferroni correction, elevated exposure to O_3_ levels at all three exposure windows were associated with elevated circulating levels of IL-1β (interleukin-1 beta), IL-8 (interleukin 8), sTNFR2 (soluble tumor necrosis factor receptor 2), and sgp130 (soluble glycoprotein 130). Elevated O_3_ at 3- and 1-month periods were associated with increased levels of sCD27 (soluble cluster of differentiation 27) and BAFF (B-cell activating factor). In pathway analyses, O_3_ was consistently and significantly associated with the pro-inflammatory/macrophage activation pathway (12-month OR = 1.49, 3-month OR = 1.57, 1-month OR = 1.54) and with B-cell activation at all three exposure windows (12-month OR = 1.24, 3-month OR = 1.53, 1-month OR = 1.41). NO_2_ was positively associated with TNFα at the 3- and 1-month exposure windows. For the PM size fractions, sporadic, mainly inverse, associations with immune markers were observed. Elevated O_3_ exposure up to one year prior to blood draw was associated with elevated immune markers related to pro-inflammatory response, macrophage activation, and B cell activation. These findings suggest potential immunologic pathways linking air pollution to adverse health outcomes in women.

## Introduction

Exposure to ambient air pollutants, including tropospheric ozone (O_3_), nitrogen dioxide (NO_2_), and particulate matter (PM; PM_0.1_ [ultrafine particles, less than 0.1μm], PM_2.5_ [fine particles, less than 2.5μm], and PM_10_ [coarse particles, less than 10μm]), has been linked to a number of adverse health outcomes, including cardiovascular and respiratory diseases^1,2^. NO_2_ is an irritant gas that is created from the atmospheric reaction of NO emitted from combustion processes^1,3,4^. According to the American Lung Association, the largest contributors of ambient NO_2_ in urban areas are transportation-related, including emissions from trucks, buses and cars^4^. NO_2_ and NO_x_ react in the presence of sunlight in the atmosphere to form secondary pollutants, such as O_3_^1,4,5^. PM is both a primary and secondary pollutant. Primary PM is directly emitted from natural sources such as wildfires and windblown dust as well as anthropogenic sources, such as combustion and resuspended road dust^3^. Secondary PM is formed from precursor gases, including NOx and volatile organic compounds (VOCs), through complex chemical reactions. Although regulatory efforts by the U.S. Environmental Protection Agency have led to declines in NO_2_, O_3_, and PM_2.5_ over recent decades^6,7^, epidemiological studies continue to report adverse health outcomes even at low ambient concentrations^1,3,8–10^.

Of particular concern for this study is the consistent evidence linking air pollutant exposure to increased cardiovascular disease (CVD) risk, particularly among post-menopausal women who face heightened risks of CVD and stroke^11,12^. Given that immune system response function also undergoes significant changes during and after menopause, understanding how air pollution influences immune responses in this population is critical for informing disease prevention efforts^13^.

The primary route of human exposure to air pollutants is through inhalation^1,3,4^. Ultrafine particles can penetrate deep into the lung and enter the bloodstream where they may trigger inflammatory response by releasing inflammatory mediators^2,8,14^. Inflammation can thus occur locally in the lungs and systemically once the pollutant enters the bloodstream. However, prior epidemiological studies of associations between air pollutants and circulating immune biomarkers have been limited in scope to interleukin-6 (IL-6), tumor necrosis factor alpha (TNFα), and interleukin-1 beta (IL-1β); prior studies generally do not include or account for multiple air pollutants and are often restricted to short-term exposures^15–23^. Moreover, studies of PM_0.1_ remain scarce, despite growing concern of their potential to elicit the greatest risk to adverse health outcomes due to their small size and ability to penetrate deep into the lung^24–27^. To address these gaps, we evaluated associations between five major air pollutants (O_3_, NO_2_, PM_0.1_, PM_2.5_ and PM_10_) and 15 immune markers and across multiple exposure windows. To our knowledge, this is the first study to shed light on pollutant-mediated adverse health outcomes; we hypothesize that exposures to pollutants would be associated with elevated inflammatory marker levels. Importantly, we cover short and long-term periods while focusing on a cohort of 1,898 women, most of whom are post-menopausal, a population shown to have higher risk compared to men for cardiovascular events such as strokes^9,14,28^ and for whom pollution-induced immune changes may have heightened clinical relevance.

## Methods

### Study population

The California Teachers study (CTS) is a prospective cohort study of 133,477 women who were active or recently retired public-school professionals in 1995 and have been followed since for health outcomes. The CTS cohort has been previously described^29^ and is approved by the Institutional Review Board of City of Hope. Participants provided informed consent at baseline. All methods were performed in accordance with relevant guidelines and regulations. In all, six questionnaires have been administered to CTS participants, and, in 2013–2016, 14,374 participated in a biobanking study (consent was obtained before collection)^30^. A subset of 1,900 of these participants were selected for inclusion in the present cross-sectional study if they had blood drawn in 2015; participants were further oversampled for the following characteristics: low SES at baseline, lived in a rural area at baseline, were not White, lived in a zip code with PM_2.5_ of 12 + μg/m^3^, or were known to live in a county that experienced a wildfire at the time of blood draw. Two participants were excluded due to missing exposure estimates.

### Immune marker measurements

From 7/1/2013 to 8/31/2016, under grant award UM1-CA164917, we collected 14,374 blood samples. Blood samples were shipped overnight to Fisher BioServices (FBS) in Rockville, MD, for processing and storage; 96% of samples were processed at FBS within 24 hours of collection (and 99.5% within 27 hours); average time from collection to processing was 18 hours.

Serum samples processed from whole blood collected were evaluated at University of California, Los Angeles for 15 immune markers by multiplexed immunometric assays (two Luminex panels; R&D Systems) and a Bioplex 200 system (Bio-Rad). Panel 1 included the detection of IL-1β, IL-6, IL-8 (interleukin-8), IL-10 (interleukin-10), and TNFα. Panel 2 included the detection of BAFF (B-cell activating factor), CCL2 (chemokine ligand 2), CCL17 (chemokine ligand 17), sCD14 (soluble cluster of differentiation 14), sCD25 (soluble cluster of differentiation 25), sCD27 (soluble cluster of differentiation 27), sCD163 (soluble cluster of differentiation 163), sgp130 (soluble glycoprotein 130), sIL6Rα (soluble interleukin 6 receptor subunit alpha), and sTNFR2 (soluble tumor necrosis factor receptor 2). These immune markers have been identified as contributors in the following immune pathways: 1) pro-inflammatory/macrophage activation: IL-1β, IL-6, sIL-6Rα, IL-8, TNFα, sTNFR2, CCL2, sCD14, sCD163, and sgp130; 2) B-cell activation: IL-10, sIL-6Rα, IL-6, sgp130, CD27, and BAFF; and 3) T-cell activation: CCL17 and sCD25. The cytokine units of measurement are in pg/ml. The laboratory assays included 5% QC replicates and duplicate samples. All assays were conducted blinded and in a single batch using the same reagent production lot, to eliminate seasonal, lot and batch variations, and were done in the same laboratory (Epeldegui lab, University of California, Los Angeles) to eliminate variation, and using single lots for all reagents^31,32^. Additionally, all immune markers were evaluated individually after finding minimal evidence of correlation among them (via Spearman coefficients) (Supplemental Table S1).

### Air pollution exposures

Air pollution exposures were estimated based on geocoded addresses of CTS participants from 2000–2018. Daily air pollution exposures were estimated at 4 km spatial resolution using the University of California at Davis / California Institute of Technology (UCD/CIT) chemical transport model^33^. Briefly, the UCD/CIT model was configured to cover regions containing more than 93% of California’s population, including over 100,000 CTS participants^9^. The UCD/CIT model can be configured to use a number of gas-phase chemical mechanisms^34^. In the current study, the SAPRC11 (Statewide Air Pollution Research Center, 2011) chemical mechanism was used to predict gas-phase pollutant concentrations based on the reliable performance of this mechanism in California^35^. Model performance statistics met the goals and criteria for Chemical Transport Model (CTM) applications^36^. Raw CTM predictions are an independent estimate of pollutant concentrations based on fundamental physics and chemistry equations that do not depend on the measured ambient values. Those estimates are based on emissions inventories developed through observations of activities that release pollutants, chemical mechanisms developed based on detailed reactions that occur in the atmosphere, transport calculations that account for advection and turbulent diffusion, dry- and wet-deposition calculations, etc. Bias in the CTM calculations can occur for various reasons, such as inaccurate wind fields/atmospheric boundary layer height, incomplete emissions estimates, etc. In the current study, we analyzed the bias in the CTM calculations through a comparison to regulatory monitors that measured PM_2.5_ mass and chemical composition. We used a Random Forest Regression (RFR) model to predict the bias in grid cells where monitoring data was not available using predicted meteorological variables (temperature, wind speed, relative humidity, etc), low-cost sensor measurements of PM_2.5_ mass, and predicted source activity based on source tracers embedded in the CTM calculations. Raw CTM corrections were corrected using the predicted bias by the RFR method. UCD/CIT estimates of daily PM total mass (PM_0.1_, PM_2.5_ and PM_10_; μg/m^3^), O_3_ (1 hour max ppm) and NO_2_ (24 hour average ppm) were assigned to the geocoded residential locations of the CTS participants for the year prior (2014) to the date of their blood draw (2015)^9,37^. From these daily estimates, different exposure windows were assigned to each participant representing 12-month (long-term), 3-month (short-term), and 1-month (short-term) averages before their blood draw date. Additionally, Pearson coefficients for the pollutants are presented in Supplemental Table S2.

### Covariates

Covariates considered included those previously associated with immune markers and/or air pollution^9,12,28,33^. The most parsimonious model was selected and included age and BMI^9,38^. For multivariable models, in addition to adjusting for age (continuous) and BMI (categorical; <25, 25–29, or 30 + kg/m^2^), O_3_ and NO_2_ models were each adjusted for all PM masses, and the PM models were similarly each adjusted for O_3_ and NO_2_. The addition of temperature (annual averaged similarly to the pollutants) into the multivariable models did not alter magnitude of risk and was not included in final models.

### Statistical analyses

Multinomial logistic regression was used to estimate associations between air pollutant exposures and immune markers (in quartiles). Quartiles were used in order to inform whether the resultant associations were dose-dependent (e.g., increasing exposure with increasing levels of cytokines) or conferred a threshold effect (e.g., increasing exposure associated after certain level or only the highest level of circulating immune markers). Because each pollutant had different units (e.g., O_3_ is measured as 1-hour max ppm whereas NO_2_ is measured as 24-hour average ppm; PMs were measured as 24-hour average μg/m3), we scaled the effect estimates by the interquartile range (IQR) to facilitate comparisons among the exposures. Results are reported as odds ratios (OR) and 95% confidence intervals (95% CI) scaled by the IQR of the pollutant. To account for multiple comparisons, Bonferroni correction was applied. Additionally, sensitivity analyses were conducted by first removing immune marker outliers (defined as ± 1.5*IQR) and rerunning the models, followed by excluding extreme pollutant values.

Immune markers were further categorized into immune pathways as: 1) pro-inflammatory/macrophage activation: elevated levels of IL-1β, IL-6, sIL-6Rα, IL-8, TNFα, sTNFR2, CCL2, sCD14, sCD163, and sgp130; 2) B-cell activation: elevated levels of IL-10, sIL-6Rα, IL-6, sgp130, CD27, and BAFF; and 3) T-cell activation: elevated levels of CCL17 and sCD25. As previously conducted, pathways were defined by first identifying those with individual immune marker levels above the respective median; of those individuals, participants with the number of elevated immune markers that exceeded the median number of total immune markers in the pathway were defined as expressing the specific immune pathway^39^. For example, if a participant had elevated levels (dichotomized as above the median) of > 3 immune markers in the B-cell activation pathway, then they were designated in the B-cell activation pathway. To be considered expressing a pro-inflammatory pathway, at least 5 of the immune markers designated in that pathway would need to be elevated (above the median) for each participant. Associations between the exposures and immune pathways (dichotomized as either expressing or not expressing the specific pathway) were also assessed via ORs and 95% CIs and adjusted by age, BMI, and other pollutants. All statistical analyses were performed using SAS 9.4 (SAS Institute Inc., Cary, NC), and data analyses were conducted via the CTS Researcher Platform^40^.

## Results

### Study population characteristics

Of the 1,898 participants, 83% were aged 50 years or older, 42% had normal BMI (< 25kg/m^2^), half reported NSAID use of > 1/week, most were non-diabetic (86%), and most did not use statins (71%) at blood draw (Supplemental Table S3). As described in the methods section, compared to the general CTS population, our study population derived from the biobanking project reflected the oversampling for more racially, geographically, and economically diverse population. Briefly, 23.3% were non-White participants and the analytic population contained a more diverse group of women; our study population SES Quartile 4 (the most advantageous group) was 28.7% compared to the full CTS cohort SES Quartile 4 of 47.2%. (Supplemental Table S3).

### Air pollutant and immune marker measurements

**Figure 1:**
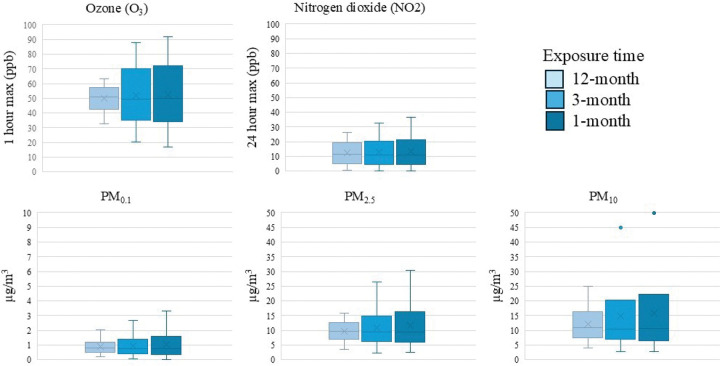
Distribution of long-term (12-month) and short-term (3-month and 1-month) averages of Ozone (O_3_; 1 hour maximum parts per billion[ppb]), Nitrogen dioxide (NO_2_; 24 hour maximum ppb), PM_0.1_ (μg/m^3^), PM_2.5_ (μg/m^3^), and PM_10_ (μg/m^3^) prior to the respective blood draw date for 1,898 women in the California Teachers Study.

Descriptive statistics for the five air pollutant exposures measured in our study are shown in Fig. 1. For visualization and consistency with other studies, estimates for O_3_ and NO_2_ were converted to ppb; however, the units of the models remain as estimated (ppm). Overall, there was relatively high consistency between exposures at the 1-month, 3-month and 12-month time periods before blood draw, particularly for O_3_ and NO_2_ where the medians were the same for the 3 exposure periods examined (O_3_ ~ 51 ppb, 1 hr max; NO_2_ ~ 12 ppb, 24 hr avg). PM exposures over the 12-months before blood draw had higher medians than 1- and 3-month exposures (PM_0.1_: 0.80μg/m^3^, PM_2.5_: 9.49μg/m^3^; PM_10_: 10.50μg/m^3^). Mean levels were largely consistent with the median, although slightly higher for PMs. Exposures averaged over the 1-month window were more variable and had wider ranges.

Quantiles and ranges of the immune markers are shown in Table 1. Detectable levels (i.e. >0 pg/mL) were observed in most participants except IL-1β, IL-6, CCL2, CCL17, and sCD163. All markers were detected at the 10th percentile.

### Air pollutant exposure associations with immune markers

**Figure 2:**
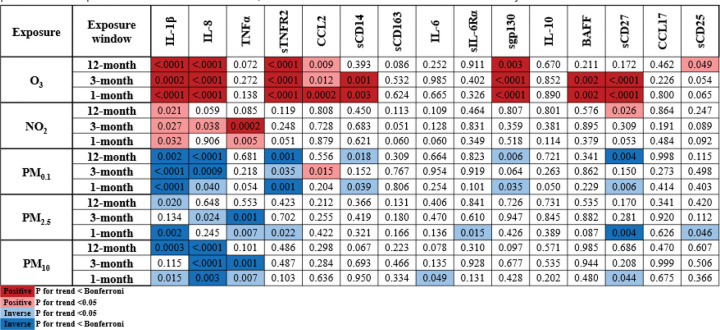
Visual of the significant p for trends for exposures (scaled by IQR for 12-month, 3-month, and 1-month prior to date of blood draw) with immune markers (quartiles). Significant associations (ORs) are denoted by color: 1) positive associations = red and pink; 2) inverse associations = dark blue and light blue; 3) p for trend value <0.05 = lighter shades; 4) p for trend value < Bonferroni correction p value = darker shades. O_3_ and NO_2_ models were adjusted for age, BMI, and all other exposures (Bonferroni correction p value=0.001). PM size fraction models were adjusted for O_3_ and NO_2_ (Bonferroni correction p value = 0.002).

The multivariable ordinal logistic regressions (scaled by IQR) showed consistent associations between O_3_ exposure and immune markers (Fig. 2, Supplemental Tables S4–8). Across all exposure windows, higher O_3_ was statistically significantly associated with increased odds of circulating IL-1β, IL-8, sTNFR2, and sgp130, with additional associations for sCD27 and BAFF at shorter exposure periods. Associations were strongest for 12-month exposure for IL-1β (OR_Quartile 4_ = 1.99, 95% CI = 1.55–2.56, p for trend < 0.0001) and IL-8 (OR_Quartile 4_=2.92, 95% CI = 2.30–3.71, p for trend < 0.0001), whereas sTNFR2, sgp130, sCD27, and BAFF showed larger effects at 3- and 1- month exposures (Fig. 2 and Supplemental Table S4).

At the pathway level (Table 2), O_3_ was consistently associated with the pro-inflammatory/macrophage activation pathway across all exposure timepoints (12-month OR = 1.49, 95% CI = 1.26–1.75; 3-month OR = 1.57, 95%CI = 1.34–1.85; 1-month OR = 1.54, 95% CI = 1.33–1.80) and for the B-cell activation pathway (12-month OR = 1.24, 95% CI = 1.05–1.47; 3-month OR = 1.53, 95% CI = 1.30–1.81; 1-month OR = 1.41, 95% CI = 1.21–1.65). Associations with the T cell activation pathway were borderline significant at the shorter time periods (3-month and 1-month).

Fewer associations were observed with NO_2_, though a notable positive association was observed with higher levels of TNFα across short term exposure windows (3-month OR = 1.63, 95%CI = 1.22–2.17; 1-month OR = 1.40, 95% CI = 1.07–1.83) (Fig. 2 and Supplemental Table S5).

PM size fractions yielded largely consistent patterns: 1) decreased IL-1β levels at all 3 time points, except a lack of significance at the 3-month period for PM_2.5_ and PM_10_, 2) decreased IL-8 levels at all 3 exposure windows for PM_0.1_ and PM_10_ but only the 3-month exposure for PM_2.5_, and 3) decreased TNFα levels for shorter exposure periods for PM_2.5_ and PM_10_ size fractions (Fig. 2 and Supplemental Tables S6–8). Lastly, the results of the sensitivity analyses were consistent with the findings from the original models.

## Discussion

In this cross-sectional analysis within the California Teachers Study, we examined the association between exposures to five air pollutants (O_3_, NO_2_, PM_0.1_, PM_2.5_ and PM_10_), estimated using a state-of-the-science chemical transport model at the participants’ residences with 15 circulating immune markers assessed from the blood samples of 1,898 participants collected in 2015. We observed two main findings: 1) higher O_3_ levels across all exposure windows (1-month to 1-year averages prior to blood draw) were associated with increased levels of several circulating immune markers linked to macrophage activation, pro-inflammatory response and B cell activation, including IL-1β, IL-8, sTNFR2, sgp130, sCD27, and BAFF; and 2) higher NO_2_ exposure was associated with increased levels of TNFα. In contrast, we note inconsistent, inverse associations between PM size fractions and immune markers, specifically IL-1β, IL-8, and TNFα.

Overall, O_3_ emerged as the most robust exposure eliciting immune responses, with positive associations across multiple immune markers. These findings align with the small number of epidemiologic studies that have linked O_3_ exposure with higher IL-1β and IL-8 levels, despite differing methods of exposure estimates and immune marker measurements^41–43^. Our study results are also consistent with prior *in vitro* and *in vivo* studies showing that O_3_ exposure increases levels of IL-8 expression^44–47^. The observed association between O_3_ exposure and increased sTNFR2 is consistent with a prior report of increased TNFR2 levels in relation to short-term (1–7 day) O_3_ exposure^48^. To our knowledge, the associations of O_3_ with sgp130, sCD27, and BAFF have not been previously reported in epidemiological studies.

Briefly, IL-1β is released by cells among the innate immune system to drive inflammatory processes^49^ while IL-8 is released by a variety of immune cells to help activate neutrophils and promote inflammation^50^. sTNFR2 is a protein that promotes T cell activity to drive pro-inflammatory processes while also suppressing immune activity by preventing TNF-induced cell death^51^. sgp130 is involved in pro-inflammatory processes by altering T cell differentiation^52^. sCD27 is a member of the TNF family and is a marker for B cell activation, specifically that of memory B cells^53,54^. Finally, and similarly to sCD27, BAFF belongs to the TNF family and plays a role in B cell maturation and survival^55^.

Our a *priori* delineation of immune pathways supported these individual marker results. Higher O_3_ levels at all 3 exposure windows was associated with pro-inflammatory/macrophage activation pathway, consistent with experimental *in vivo* and *in vitro* studies showing that O_3_ induces pro-inflammatory gene expression and the subsequent release of inflammatory markers^44,45,56,57^. Notably this pathway encompasses immune markers regulated by the NFkB pathway, a pro-central mechanism linking air pollution to cardiovascular disease risk through increased thrombosis and atherosclerosis^58–61^. Our results also suggested a link between O_3_ exposure and the B-cell activation pathway, driven by sgp130, sCD27, and BAFF. These novel findings are not yet supported in the current literature and warrant replication in other studies.

The significant associations with IL-1β in our findings are noteworthy due to its established role as an inflammatory mediator in atherosclerotic cardiovascular disease (ASCVD), including stroke and CVD. In a large, randomized, double-blind trial, an IL-1β antagonist drug, canakinumab, was reported to reduce recurrent cardiovascular events. These findings in conjunction with our reported findings present the possibility that those who are exposed to higher levels of ambient air pollutants, particularly O_3_, may benefit from pharmaceutical interventions targeting IL-1β to reduce risk of ASCVD^62^.

We also report associations between increasing NO_2_ exposure across all 3 exposure windows with higher TNFα levels. These results complement current evidence of *in vivo* and *in vitro* studies that show a relationship between elevated TNFα and NO_2_ exposure^63^.

Our results for PM exposures were contrary to what was expected. Across PM size fractions, there were decreased risk of elevated IL-1β and IL-8 levels at the different exposure windows, and short-term exposure to PM_2.5_ and PM_10_ were associated with decreased TNFα. These findings contradict prior work that have shown that exposure to PM is associated with elevated levels of IL-1β and TNFα^16,17^. It is possible, however, that the discrepant results may be due to the population subset evaluated. Prior reports have suggested associations between PM_10_ with elevated levels of IL-1β in men, but not women^16,17^; moreover, associations with IL-1β, IL-6 and TNF-α were demonstrated in younger populations, versus our population of largely post-menopausal women where baseline inflammation levels may already be higher^16,17^.

Major strengths of our study include the use of multiplex immune marker assays and the use of a state-of-the-science chemical transport model for exposure assessment. Limitations include the cross-sectional design, which precludes establishing temporal relationships, and reliance on residential address-based exposures, which may not capture exposures from work and/or travel. Our study was also nested in an established cohort (CTS) that was originally designed to investigate breast cancer, restricting our evaluation to women. Although this design was intentional for interrogating a population subset and age range that encompasses postmenopausal women, we recognize our results may not be necessarily generalizable to males and other populations^64,65^.

In conclusion, we found consistent evidence that O_3_ exposure is associated with elevated levels of immune markers in both the pro-inflammatory/macrophage activation and B cell activation pathways, with exposures averaged from 1-month to one-year prior to blood draw conferring risk. For certain immune markers, such as IL-1β, TNFα and IL-8, however, associations varied by exposure window, suggesting potential critical periods of susceptibility. Replication in diverse populations and study designs will be essential to clarify the temporal and biological dynamics underlying these associations, and to better understand their role in mediating air pollution-related health outcomes.

## Supplementary Material

Supplementary Files

This is a list of supplementary files associated with this preprint. Click to download.

• AirpollcytokinesupplementalscientificreportsFINAL.docx

## Figures and Tables

**Table 1:** Distribution of measured cytokines (pg/ml) in the serum of 1,898 women in the California Teachers Study (collected in 2015).

Variable	Full variable name	Min^a^	25th^b^	Median	75th^b^	Max^c^
**IL-1β**	Interleukin-1 beta	0	1.14	1.94	3.18	26,830.31
**IL-6**	Interleukin-6	0	1.98	3.09	4.54	35,605.13
**IL-8**	Interleukin-8	0.26	14	18.99	27.08	69,406.81
**IL-10**	Interleukin-10	0.03	0.95	1.55	2.54	502.16
**TNFα**	Tumor necrosis factor alpha	1.31	14.51	19.33	24.8	1,414.93
**BAFF**	B-cell activating factor	350.87	638.86	723.93	825.63	2,747.91
**CCL2**	C-C motif chemokine ligand 2	0	359.69	416.08	486.11	14,372.14
**CCL17**	C-C motif chemokine ligand 17	0	761.72	898.32	1,101.71	20,197.32
**sCD14**	Soluble cluster of differentiation 14	226,516.77	855,261.99	941,781.34	1,030,400.00	2,222,100.00
**sCD25**	Soluble cluster of differentiation 25	209.99	453.59	551.86	680.01	2,751.44
**sCD27**	Soluble cluster of differentiation 27	2,649.49	5,350.39	6,305.89	7,677.47	68,915.68
**sCD163**	Soluble cluster of differentiation 163	0	423,469.16	535,244.81	677,261.24	3,129,200.00
**sgp130**	Soluble glycoprotein 130	45,813.76	150,910.60	169,374.84	188,790.38	400,817.39
**sIL-6Rα**	Soluble interleukin-6 receptor subunit alpha	25,274.42	46,918.78	56,027.03	65,089.76	148,465.14
**sTNFR2**	Soluble tumor necrosis factor receptor 2	529.69	1,673.25	2,028.20	2,526.87	278,416.25

a Min=minimum.

b 10th, 25th, 75th and 90th respective percentiles.

c Max=maximum.

**Table 2:** Associations between immune marker pathways and ozone (O_3_) exposures 12-months, 3-months and 1-month before blood draw.

Immune Pathway	Exposure Time^d^	Odds ratio (OR)	95% CI
Pro-inflammatory/macrophage activation^a^	12-month prior	1.49	1.26–1.75
	3-month prior	1.57	1.34–1.85
	1-month prior	1.54	1.33–1.80
B cell activation^b^	12-month prior	1.24	1.05–1.47
	3-month prior	1.53	1.30–1.81
	1-month prior	1.41	1.21–1.65
T cell activation^c^	12-month prior	1.02	0.85–1.21
	3-month prior	1.19	1.00–1.42
	1-month prior	1.17	1.00–1.38

a Pro-inflammatory/macrophage activation: elevated levels of IL-1β, IL-6, sIL-6Rα, IL-8, TNFα, sTNFR2, CCL2, sCD14, sCD163, and sgp130

b B-cell activation: : elevated levels of IL-10, sIL-6Rα, IL-6, sgp130, CD27, and BAFF

c T-cell activation: elevated levels of CCL17 and sCD25

d IQR values: 12-month = 0.0095, 3-month = 0.0169, 1-month = 0.0259

All models adjusted for age, BMI, and odds ratios are scaled by the IQR

## Data Availability

The data used in the current study are available for research use. The California Teachers Study welcomes all inquiries. Please visit https://www.calteachersstudy.org/for-researchers.
